# Selection of Reliable Reference Genes for Gene Expression Studies on *Rhododendron molle* G. Don

**DOI:** 10.3389/fpls.2016.01547

**Published:** 2016-10-18

**Authors:** Zheng Xiao, Xiaobo Sun, Xiaoqing Liu, Chang Li, Lisi He, Shangping Chen, Jiale Su

**Affiliations:** Institute of Horticulture, Jiangsu Key Laboratory for Horticultural Crop Genetic Improvement, Jiangsu Academy of Agricultural SciencesNanjing, China

**Keywords:** *Rhododendron*, qRT-PCR, flower, reference gene, normalization

## Abstract

The quantitative real-time polymerase chain reaction (qRT-PCR) approach has become a widely used method to analyze expression patterns of target genes. The selection of an optimal reference gene is a prerequisite for the accurate normalization of gene expression in qRT-PCR. The present study constitutes the first systematic evaluation of potential reference genes in *Rhododendron molle* G. Don. Eleven candidate reference genes in different tissues and flowers at different developmental stages of *R. molle* were assessed using the following three software packages: GeNorm, NormFinder, and BestKeeper. The results showed that *EF1-*α (elongation factor 1-alpha), *18S* (18s ribosomal RNA), and *RPL3* (ribosomal protein L3) were the most stable reference genes in developing rhododendron flowers and, thus, in all of the tested samples, while tublin (*TUB*) was the least stable. *ACT5* (actin), *RPL3*, *18S*, and *EF1-*α were found to be the top four choices for different tissues, whereas *TUB* was not found to favor qRT-PCR normalization in these tissues. Three stable reference genes are recommended for the normalization of qRT-PCR data in *R. molle*. Furthermore, the expression profiles of *RmPSY* (phytoene synthase) and *RmPDS* (phytoene dehydrogenase) were assessed using *EF1-*α, *18S*, *ACT5*, *RPL3*, and their combination as internals. Similar trends were found, but these trends varied when the least stable reference gene *TUB* was used. The results further prove that it is necessary to validate the stability of reference genes prior to their use for normalization under different experimental conditions. This study provides useful information for reliable qRT-PCR data normalization in gene studies of *R. molle*.

## Introduction

Rhododendrons, which are commonly referred to as rosage, are decorative shrubs with beautiful flowers that are widespread around the world in countries characterized by mild climates (Chosson et al., 1998). While there is a remarkably broad range of rhododendron flower colors, such as red, pink, purple, and white, there is no yellow-flowered cultivar of evergreen rhododendron (Ureshino et al., 2016). The enhancement of yellow flowers is one of the most important breeding objectives in regards to evergreen rhododendrons. *Rhododendron molle* is a deciduous *Rhododendron* species with unique yellow flowers. Variations in the morphological characteristics and pigments of wild evergreen rhododendron flowers have been studied in detail (Miyajima et al., 2000; Nakatsuka et al., 2008; Mizuta et al., 2009; Ureshino et al., 2016). However, factors that regulate the flower pigmentation of *R. molle* are still unknown. Therefore, the study of expression patterns of some key genes involved in flower development at the molecular level will help improve our ability to breed desirable yellow-flowered evergreen rhododendrons.

Methods for detecting gene expression levels include the following: semi-RT-PCR, Northern blot, RNase protection analysis, *in situ* hybridization and quantitative real-time polymerase chain reaction (qRT-PCR). qRT-PCR is considered to be an efficient, sensitive and reliable technique for conducting simultaneous measurements of selected gene expressions in many different samples (Galli et al., 2015; Machado et al., 2015). Compared to conventional methods, qRT-PCR is the only method available for detecting mRNA levels of low copy number target genes of interest (Huggett et al., 2005). However, qRT-PCR involves the use of appropriate normalization methods to ensure reliable measurements of target gene expressions. One of the most popular methods used to normalize qRT-PCR data involves selecting optimal reference genes, which can control potential experimental errors. Ideal reference genes should be stable at the expression level under various experimental conditions (Banda et al., 2008). Generally speaking, genes that play key roles in the maintenance of basic cellular functioning are typically selected as reference genes [e.g., Tublin (*TUB*), elongation factor 1-α (*EF1-*α), glyceraldehydes-3-phosphate dehydrogenase (*GAPDH*), actin (*ACT*), and 18s ribosomal RNA (*18S*)]. The expression patterns of reference genes can vary among different species or for the same plant under different experimental conditions. For example, *EF1-*α is the most suitable reference gene among different tissues of Chinese cabbage (Qi et al., 2010), but it is not recommended as a suitable reference gene of *Arabidopsis* (Czechowski et al., 2005). Moreover, *GAPDH* was found to be the best reference gene according to Chinese cabbage experiments conducted under conditions of drought stress (Qi et al., 2010). It is thus absolutely vital to validate the stability of reference genes before selecting them for the normalization of qRT-PCR data under specific experimental conditions.

In *R. simsii* hybrids, *GAPDH* and maturase K have been used as reference genes to flower development (De Keyser et al., 2004). However, the stability of both genes has not been verified. Moreover, a few reference genes have been validated for flower petals of azalea due to limited sequence data available (Keyser et al., 2013). Validated qualitative RT-PCR protocols for *R. molle* are still rare. Thus, the identification of reliable reference genes for gene expression normalization will facilitate further studies on *R. molle* flower development and different tissues at the transcript level. In this study, the stability of eleven potential reference genes was identified in developing flowers and *R. molle* tissues. The expression profiles of eleven reference genes including *ACT5*, *ACT7*, *EF1-*α, elongation factor 1-gamma (*EF1-*γ), *18S*, *GAPDH*, ubiquitin-conjugating enzyme E2 (*UBC*), *TUB*, NAC domain-containing protein (*NAC*), fatty acid desaturase (*FAD*), and ribosomal protein L3 (*RPL3*) were studied in seven different tissues and during six flower developmental stages. Expressions of these genes were evaluated using GeNorm (Vandesompele et al., 2002), BestKeeper (Pfaffl et al., 2004), and NormFinder (Andersen et al., 2004). Moreover, *R. molle* phytoene synthase (*RmPSY*) and *R. molle* phytoene dehydrogenase (*RmPDS*) are the main genes that control the biosynthesis of carotenoid content, which is involved in flower yellow biosynthesis. Expression patterns of *RmPSY* and *RmPDS* were investigated using the selected references, which may serve as a valuable resource for future association studies aimed at better understanding molecular mechanisms that drive flower pigmentation in *R. molle*.

## Materials and Methods

### Plant Materials

*Rhododendron molle* was obtained from a *Rhododendron* garden managed by the Jiangsu Academy of Agricultural Sciences. Seven different tissues, including flowers, new leaves, mature leaves, petioles, roots, stems and seeds were collected, frozen immediately in liquid nitrogen and stored in a -80°C freezer prior to use. Samples of flowers at six developmental stages were collected in 2016 as follows: March 9 (flower buds 2–5 mm in diameter), March 12 (flower buds 5–10 mm in diameter), March 15 (unfold-petal stage), March 18 (initial-flowering stage), March 21 (full-flowering stage), and March 24 (flower-wilting stage).

### RNA Isolation and Reverse Transcription

Total RNA was extracted using an EASYspin plus Plant RNA out RN28 kit (Ailab Technology Company, Beijing, China). The integrity of total RNA was determined by denaturing 1.0% agarose gel electrophoresis. RNA concentrations were determined using a Nanodrop 1000 spectrophotometer (Thermo, Wilmington, DE, USA). First strand cDNA synthesis was performed using a PrimeScript 1st Strand cDNA Synthesis Kit (TaKaRa, Dalian, China). The cDNA products were diluted 20 times.

### Primer Design and PCR Conditions

Two target genes and eleven reference genes were selected from the transcriptome of *R. molle*. The gene sequences are stored in GenBank (**Table 1**). Primers of the eleven reference genes and two target genes were designed through the Primer 3 program^1^. qRT-PCR was performed on cDNA, and their products were analyzed via electrophoresis on 2.0% agarose gel to determine primer features.

**Table 1:** Candidate reference genes and different parameters derived from the qRT-PCR analysis.

Gene symbol	GeneBank accession	Primer sequence (5′→3′; forward/reverse)	Amplicon length (bp)	Amplification efficiency	*R*^2^
*ACT7*	KX230458	GCAGCCAAGCCGAAGAAGAA/CTCGCTTTGCCACACTCACT	241	96.99%	0.995
*UBC*	KX230451	AACCAGAGCCCTCAAGATGC/GCAACAGTAAGCCCAACAGC	236	95.83%	0.993
*GAPDH*	KX230455	CTCGCTACCAGATGTGCCAA/ATGCTCCTCTTGTGTCGGTG	220	96.02%	0.979
*TUB*	KX230460	CGTTGATCATCTGCTCGTCG/CGCGTCTCCACTTCTTCATG	219	98.11%	0.993
*ACT5*	KX230452	CGGTGCCCTGAAATCCTGTT/AAACGCTCAGCCATTCCAGG	218	95.76%	0.969
*RPL3*	KX230459	CATCTGAGCGAGGAGGTGAA/GGTGTGCCTTCTTCTGCTTC	217	96.64%	0.975
*18S*	KX230463	AGGGATGACTTGGAGCGACTG/CTGAAAGAAGTGCTGATGGTGG	215	106.89%	0.998
*NAC*	KX230457	TGGAACTCGGAAGCGTAGAA/TTCTCCAGTGCCCAAGTGAT	205	94.24%	0.981
*FAD*	KX230456	GCCTCTTTATTGGGCTGCTC/CGTGGTTTTGATGGTGGGTT	188	104.62%	0.955
*EF1-*γ	KX230454	AAGCGAGAAACATAGCGTGC/TCAAGACACTCATTGCTGCG	187	96.64%	0.999
*EF1-*α	KX230453	AGATGATTCCGACCAAGCCT/TTGGCAGCAGACTTTGTGAC	168	96.29%	0.998
*RmPSY*	KX230461	GTCTCCCCATCAAGAACGTCG/CATGTGAATAAGTTGTGGCCCTC	229	95.72%	0.998
*RmPDS*	KX230462	ACGAATTGCTTGCTTCCCG/GCACTCTTAGGGATTCGCTGTC	216	96.49%	0.997

Quantitative real-time polymerase chain reaction reactions were performed using a 7,500 Real-Time PCR system (Applied Biosystems, Carlsbad, CA, USA). In a white 96-well plate (Axygen, Union City, CA, USA), 10 μL of SYBR Premix Ex Taq (TaKaRa, Dalian, China) and 0.4 μL of 10 μM forward primer and reverse primer was used in combination with 2 μL of cDNA, resulting in a total volume of 20 μL. Cycling conditions involved 10 s at 95°C followed by 40 cycles of 5 s 95°C and 34 s 60°C. A melting dissociation curve was created to identify amplicon characteristics. The final threshold cycle values were average values. Each reaction was performed in triplicate.

### Gene Stability Analysis

Standard curves were used to determine the gene-specific PCR efficiency level from a 10-fold dilution of the mixed cDNA template for each primer pair. Correlation coefficients (*R*^2^) and slope values were obtained from the standard curve, and corresponding PCR amplification efficiencies (*E*) were determined according to the following equation: *E* = (10^-1/slope^-1) × 100 (Radonić et al., 2004).

Reference gene expression stability was statistically analyzed based on the results obtained from the following three Excel-based software programs: GeNorm (Vandesompele et al., 2002), NormFinder and BestKeeper (Andersen et al., 2004; Pfaffl et al., 2004). The GeNorm program was used to calculate the expression stability value (*M*) for reference genes based on the pairwise variation. Stable expression genes were characterized with lower *M* values. The BestKeeper program uses an index for the evaluation of expression stability that is calculated based on standard deviation (SD) and percentage covariance (CV) values (Sang et al., 2013). The NormFinder program can determine degrees of variance within and between groups, and the gene with the lowest stability value is ranked as the best.

## Results

### Primer Specificity, RNA Integrity, Reference Gene Candidate, and PCR Amplification Efficiency Verification

**FIGURE 1:**
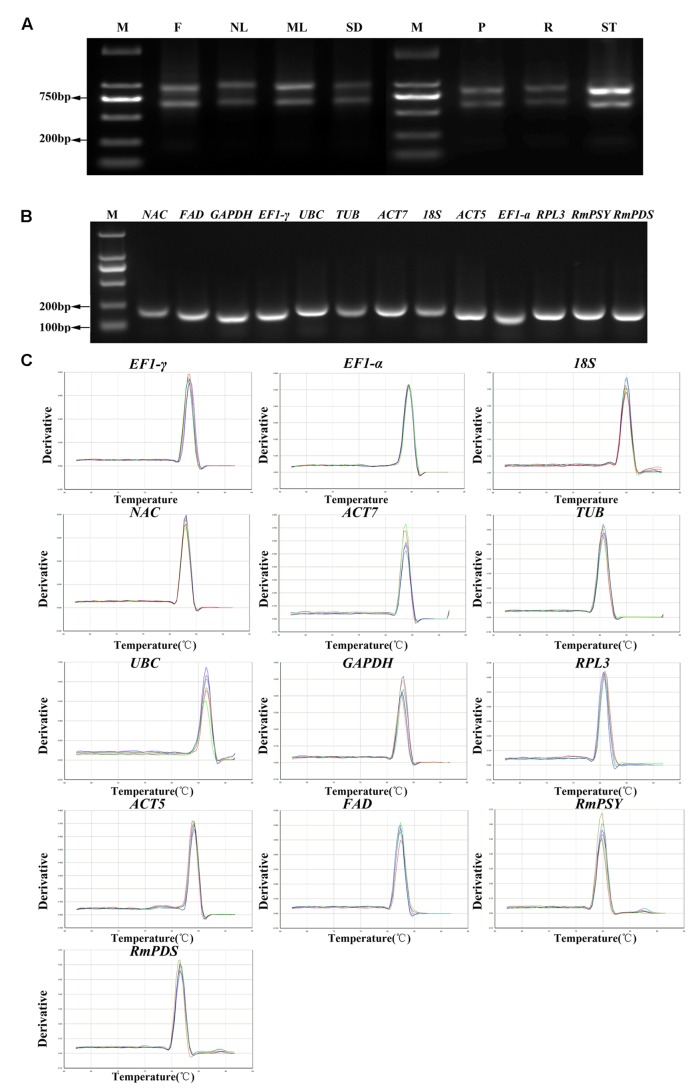
**RNA extraction, confirmation of gene specificity and amplicon size. (A)** The integrity of RNA samples were determined by electrophoresis on agarose gel. F, flowers; NL, new leaves; ML, mature leaves; SD, seeds; P, petioles; R, roots; ST, stems. **(B)** Agarose gel showing qRT-PCR products for each gene with the expected size. **(C)** Melting curves of 13 genes with a single peak. M denotes the 2,000 bp DNA marker.

Eleven reference genes were selected from the transcriptome of *R. molle* and two other genes were obtained (**Table 1**). High quality of the RNA samples is critical for a successful qRT-PCR. The results showed that ratio value of 260/280 nm wavelength between 1.8 and 2.1, and 260/230 nm wavelength higher than 2.0. The degradation of the RNA was minimal (**Figure 1A**). Melting temperatures (*Tm*) of all of the PCR products ranged from 81.2°C for *RPL3* to 86.7°C for *UBC*. The amplification efficiency of PCR reactions varied from 94.24% for *NAC* to 106.89% for *18S*, and correlation coefficients (*R*^2^) ranged from 0.955 for *FAD* to 0.999 for *EF1-*γ (**Table 1**). Amplifications were confirmed based on the presence of single fragments of an expected size during 2.0% agarose gel electrophoresis (**Figure 1B**). Furthermore, only one distinctive peak was found in the melting curves, indicating that no primer dimmers were generated from non-specific amplification (**Figure 1C**).

**FIGURE 2:**
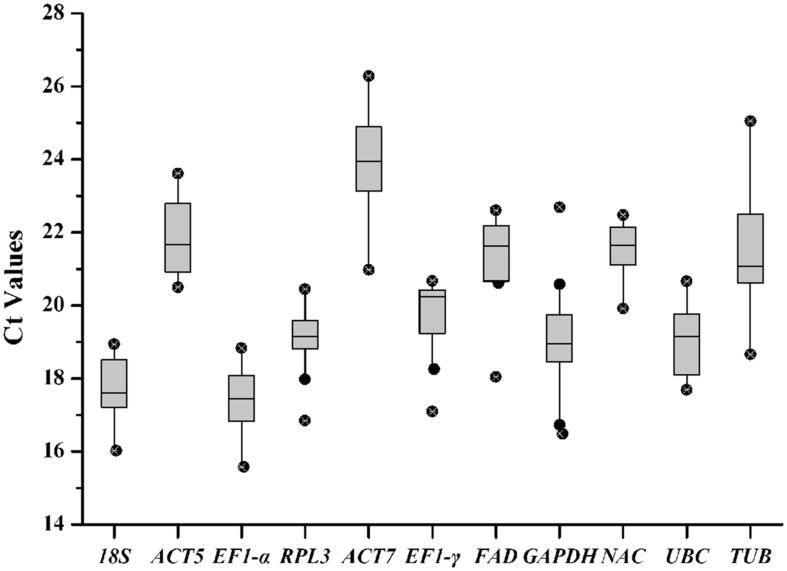
**Quantitative real-time polymerase chain reaction Ct values for reference genes.** Ct values for each reference gene in all of the *R. molle* samples. A line across the box denotes the median. The box denotes the 25 and 75% percentiles, whisker caps represent maximum and minimum values, and dots represent outliers.

Cycle threshold (Ct) values were calculated to determine transcript levels of the reference gene in the tested samples. The eleven reference genes exhibited a relatively broad degree of transcript level dispersal. Average Ct vales ranged from 15.57 for *EF1-*α to 26.28 for *ACT7* (**Figure 2**). *EF1-*α with narrow variance was found to be the most common reference transcript and *ACT7* was identified as the least common. The coefficient of variance (CV) of Ct values can determine reference gene stability levels. *GAPDH* presented a high degree of transcript level variation with a CV value of 8.59% while *NAC* presented the lowest degree of variation with a CV value of 3.57%.

### GeNorm Analysis

**FIGURE 3:**
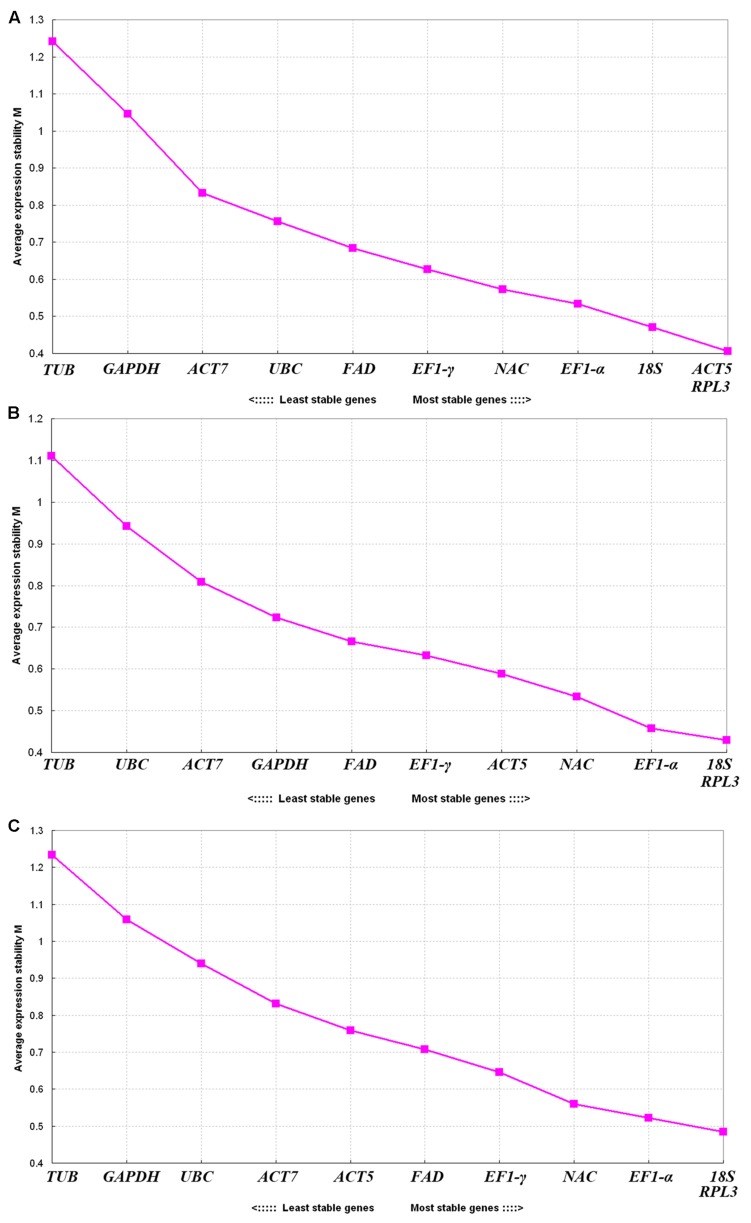
**Expression stability values (*M*) of eleven reference genes calculated through GeNorm. (A)** Different tissues, **(B)** flower samples of different developmental stages, and **(C)** all samples. Lower average expression stability levels (*M* value) denote more stable expression.

GeNorm was used to select an ideal pair of reference genes by calculating gene expression stability values *M* based on the average pairwise expression ratio. Genes with low *M* values were characterized as the most stable reference genes (Vandesompele et al., 2002). The program guidelines recommend using *M* values falling below a threshold of 1.5 to identify ideal reference genes with stable expression. Our analysis showed that the *M* values of all eleven genes were lower than 1.5, indicating that they all conformed to basic requirements for the reference gene (**Figure 3**). *ACT5* and *RPL3* were the most stable genes found in the seven tissues with the lowest *M* value (0.40), and *TUB* was the least stable gene with the lowest *M* value (1.24) (**Figure 3A**). For the flower samples representing six different developmental stages, *18S* and *RPL3* showed the highest levels of expression stability and the lowest *M* value (0.42), and *TUB* was the least stable gene with the lowest *M* value (1.11) (**Figure 3B**). Meanwhile, *18S* and *RPL3* were found to be the most stable genes, and *TUB* was found to be the least stable gene in all of the samples (**Figure 3C**). Overall, *RPL3*, *18S*, *EF1-*α, and *ACT5* showed relatively higher degrees of stability with lower *M* values while *TUB* presented the lowest levels of stability.

**FIGURE 4:**
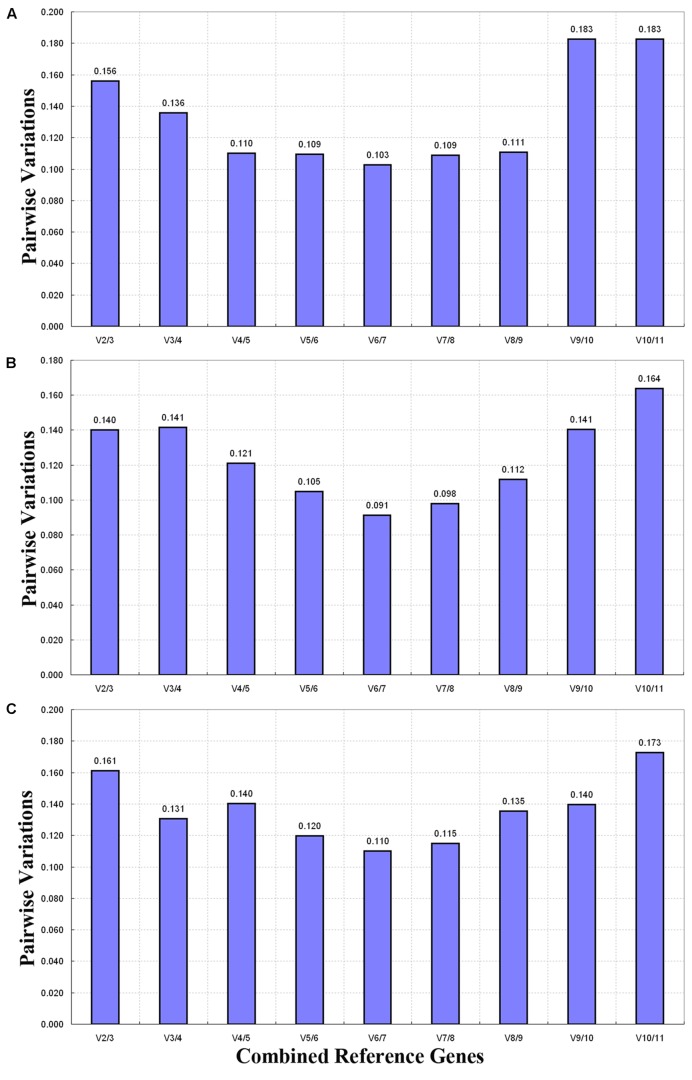
**Pairwise variation (V) calculated through GeNorm to determine the optimal number of reference genes.** The average pairwise variation (*V*_n_/*V*_n+1_) between normalization factors NF_n_ and NF_n+1_was analyzed to determine the optimal number of reference genes required for qRT-PCR data normalization in different samples. **(A)** Different tissues, **(B)** flower samples of different developmental stages, and **(C)** all samples.

The optimal number of reference genes was determined by determining the pairwise variation (*V*_n_/*V*_n+1_) between two sequential normalization factors (NF_n_ and NF_n+1_) through GeNorm (**Figure 4**). Generally speaking, 0.15 was used as a cutoff value to determine the optimal number of reference genes (Vandesompele et al., 2002; Pfaffl et al., 2004). In seven tissue sample sets, the paired variable coefficient for different tissues showed that two stable genes (*V*_2/3_ > 0.15) were deficient in determining variations of the normalization factor, whereas three stable reference genes (*ACT5*, *RPL3*, and *18S*; *V*_3/4_ < 0.15) were found to be sufficient for normalizing gene expression (**Figure 4A**). When the flower samples of six developmental stages were tested, all pairwise variations except for *V*_10/11_ (0.164) fell below the cutoff value (0.15) (**Figure 4B**), thus indicating that one reference gene was sufficient for normalization. For all of the samples, *V*_2/3_ (0.161) was higher than the given threshold (0.15), and the other pairwise variations except for *V*_10/11_ (0.173) were lower than 0.15 (**Figure 4C**), thus showing that a third internal gene was required to normalize gene expression.

### NormFinder Analysis

NormFinder ranks candidate genes based on the average pairwise variation in a gene relative to other candidate genes (Han et al., 2012). The stable gene generated lower average expression stability values (Andersen et al., 2004). Gene stability values were determined using NormFinder as shown in **Table 2**. *ACT5* and *RPL3* were ranked by NormFinder as the most stable genes in the seven tissues (**Table 2**). *EF1-*α and *RPL3* were ranked as the most stable genes in all of the samples owing to their low stability values. This result is consistent with the results determined through GeNorm. When considering the flower samples of six developmental stages, the stability ranking of the eleven candidate genes drawn from the NormFinder program showed somewhat different values from those drawn from GeNorm as follows: *EF1-*α and *ACT5* were ranked consistently through GeNorm, whereas *RPL3* and *18S* were omitted from the list of most stable genes. Like the GeNorm program, NormFinder characterized *TUB* as the least stable gene.

**Table 2:** Ranking of candidate reference genes by expression stability levels determined via NormFinder.

Rank	Tissues		Different developing flowers		Total	
	Gene name	Stability value	Gene name	Stability value	Gene name	Stability value
1	*ACT5*	0.114	*EF1-à*	0.114	*EF1-à*	0.218
2	*RPL3*	0.141	*ACT5*	0.169	*RPL3*	0.270
3	*18S*	0.216	*EF1-*γ	0.280	*NAC*	0.346
4	*EF1-à*	0.298	*NAC*	0.291	*EF1-*γ	0.371
5	*FAD*	0.397	*FAD*	0.416	*18S*	0.411
6	*NAC*	0.419	*RPL3*	0.444	*ACT5*	0.435
7	*EF1-*γ	0.465	*GAPDH*	0.487	*FAD*	0.435
8	*UBC*	0.608	*18S*	0.526	*ACT7*	0.725
9	*ACT7*	0.664	*UBC*	0.832	*UBC*	0.750
10	*GAPDH*	1.314	*ACT7*	0.861	*GAPDH*	0.993
11	*TUB*	1.381	*TUB*	1.238	*TUB*	1.306

### BestKeeper Analysis

BestKeeper is used to evaluate the expression stability of reference genes based on the coefficient of correlation (r) of the BestKeeper index based on SD and CV values (Pfaffl et al., 2004). The BestKeeper analysis showed that the best correlations were obtained for *EF1-*α (0.936), *FAD* (0.919), and *RPL3* (0.909) for all of the samples (**Table 3**). For the different tissues, *EF1-*α (0.914), *RPL3* (0.902), *18S* (0.843), and *ACT5* (0.799) showed the highest correlations, which is consistent with results drawn from GeNorm and NormFinder. When considering the flower samples of different growth phases, BestKeeper analyses revealed few differences among results produced by the three programs. Overall, *EF1-*α showed a strong correlation with the BestKeeper index in all of the experimental sets consistent with the corresponding GeNorm and NormFinder results. Therefore, *EF1-*α is recommended as the ideal reference gene for normalization.

**Table 3:** Expression stability values of ten selected genes determined through BestKeeper.

Rank	Tissues			Different developing flowers			Total		
	Gene name	Coefficient of correlation (r)	*p*-value	Gene name	Coefficient of correlation (r)	*p*-value	Gene name	Coefficient of correlation (r)	*p*-value
1	*EF1-*α	0.91	0.004	*FAD*	0.99	0.001	*EF1-*α	0.94	0.001
2	*RPL3*	0.90	0.005	*EF1-*α	0.99	0.001	*FAD*	0.92	0.001
3	*18S*	0.84	0.017	*EF1-*γ	0.95	0.003	*RPL3*	0.91	0.001
4	*ACT5*	0.80	0.031	*ACT5*	0.95	0.004	*EF1-*γ	0.85	0.001
5	*FAD*	0.79	0.034	*NAC*	0.94	0.006	*18S*	0.81	0.001
6	*NAC*	0.62	0.139	*ACT7*	0.93	0.007	*ACT7*	0.79	0.001
7	*EF1-*γ	0.62	0.141	*RPL3*	0.92	0.010	*NAC*	0.76	0.003
8	*GAPDH*	0.59	0.168	*GAPDH*	0.88	0.021	*ACT5*	0.73	0.005
9	*UBC*	0.51	0.241	*18S*	0.83	0.040	*GAPDH*	0.66	0.013
10	*ACT7*	0.42	0.350	*UBC*	0.33	0.523	*UBC*	0.35	0.241

### Reference Gene Validation

**FIGURE 5:**
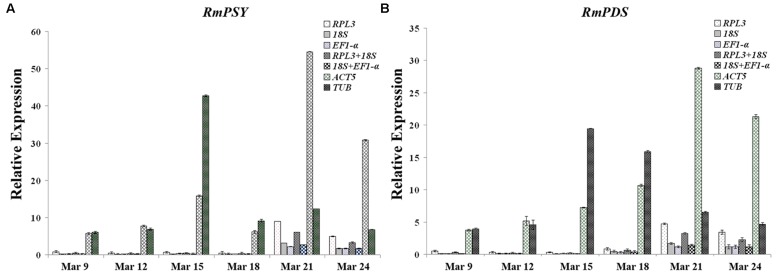
**Expression levels of *RmPSY* and *RmPDS* throughout the flower development of *R. molle*. (A)** Expression levels of *RmPSY* at different stages of flower development. **(B)** Expression levels of *RmPDS* at different stages of flower development. Genes were normalized to different reference genes. Error bars show the standard error calculated from three biological replicates.

Optimal reference genes should have a significant effect on final normalized results. To determine the effects of reference genes on experimental results and to validate the stability of reference genes, expression patterns of two functional genes (*RmPSY* and *RmPDS*) occupying different stages of flower development were evaluated using different reference genes (*EF1-*α, *ACT5*, *18S*, and *RPL3*). The expression patterns of *RmPSY* and *RmPDS* showed low levels of expression during earlier stages of flower development (March 9 and 12) and reached to maximum expression levels during later stages (March 21). The expression profiles of the two target genes showed similar trends when stable reference genes were used (*EF1-*α, *ACT5*, *18S*, and *RPL3*) (**Figure 5**). When *TUB* was used as an internal gene, the highest expression level of the target genes was reached at an earlier stage (March 15), contradicting the other stable reference gene results. This showed that the least stable gene *TUB* failed to standardize the expression data effectively.

## Discussion

The quantitative real-time PCR approach has become a key method of gene expression profiling owing to its accuracy, sensitivity, and efficiency (Jacobsen et al., 2016; Sgamma et al., 2016; Song et al., 2016). It is crucial for selecting reference genes that are stably expressed amongst treatment groups in real-time quantitative PCR gene expression studies. A good reference gene should maintain invariable expression levels in different tissues, organs and developmental stages and under various experimental conditions (Chapman and Waldenström, 2015). Therefore, the selection of reference genes that are stably expressed across different samples can improve the accuracy and reliability of results obtained through qRT-PCR (Radonić et al., 2004).

The GeNorm program was used to determine the stability of a candidate gene by pairwise comparison, and the NormFinder and BestKeeper programs were used to prevent co-regulation and to further assess the analysis results obtained from the GeNorm program (Cruz et al., 2009; Zhao et al., 2011). From the GeNorm evaluation, we found that *RPL3*, *18S*, and *EF1-*α are the most stable reference genes in all of the samples examined (**Figure 3**). Ribosomal genes are often found to serve as good housekeeping genes, as they carry out the biogenesis of new ribosomes and are expressed in all cells (Hsiao et al., 2001). Thus, *RPL3*, which encodes ribosomal protein L3, is known to be the most reliable gene in seedlings and anther-regenerated plants of different ploidy of *Dendrocalamus latiflorus Munro* (Liu et al., 2014). *EF1-*α also presents stable expression patterns in water lily tissues (Luo et al., 2010) darnel ryegrass (Martin et al., 2008), grape berries (Reid et al., 2006), and cucumbers (Wan et al., 2009). Moreover, *EF1-*α shows stable expression in potato and soybean plants (Nicot et al., 2005; Jian et al., 2008). However, *EF1-*α is not recommended as a suitable reference gene in *Arabidopsis* and Moso bamboo (Czechowski et al., 2005; Fan et al., 2013). In addition, *18S*, *ACT*, *TUB*, and *GAPDH* are commonly used as traditional reference genes in qRT-PCR assays (Niu et al., 2015; Reddy et al., 2015). *18S* is the most suitable stability gene for different organs and growth stages (Xu et al., 2016), but it is less stable in broomrape tissues (González-Verdejo et al., 2008) and peaches (Tong et al., 2009). *ACT7* presents the most stable expression across pear (Xu et al., 2015) and tung samples that have been examined (Han et al., 2012), but it is not suitable as a reference gene in *A. thaliana* (Remans et al., 2008). *GAPDH* and *ACT11* are the two top-ranked reference genes in seedlings of *Panax ginseng* treated with heat (Wang and Lu, 2016). In the present study, *ACT7*, *GAPDH*, and *TUB* were found to be unstable. *18S* and *ACT5* were also ranked first in terms of stable reference genes across all of the *R. molle* samples examined. These results show that reference genes should be reconfirmed according to experimental conditions or plant species.

One reference gene is typically used in qRT-PCR data normalization (Suzuki et al., 2000). However, it has been insufficient to select only one reference gene in some gene expression studies (Veazey and Golding, 2011). GeNorm guidelines recommend that a cutoff value of 0.15 is used to determine the optimal number of reference genes (Han et al., 2012). In the three experiment sets, all pairwise variations of *V*_3/4_ fell below 0.15 (**Figure 4**). This shows that a combination of three reference genes is sufficient for the normalization of qRT-PCR data obtained from different tissues of *R. molle* or flower samples.

*Rhododendron molle* phytoene synthase and *RmPDS* were found to be the main genes controlling the biosynthesis of carotenoid content and were found to be involved in the flower yellow biosynthesis of *R. molle* (Nisar et al., 2015). To validate the suitability of the reference genes, the expression profiles of *RmPSY* and *RmPDS* were assessed in the different flower samples using*EF1-*α, *18S*, *RPL3*, *ACT5, TUB*, and a combination of these as internal controls. As a result, *RPL3*/*18S* and *18S*/*EF1-*α were selected as optimum pairs of reference genes for qRT-PCR data normalization for different flower samples of *R. molle*. The expression profiles of *RmPSY* and *RmPDS* showed low degrees of expression in earlier stages of flower development (March 9 and 12) and reached maximum expression levels during later stages (March 21). The expression patterns of the two target genes showed similar trends when stable reference genes *EF1-*α, *18S*, and *RPL3* or a combination of stable reference genes were used (**Figure 5**). The relative transcript abundance presented conflicting results when the least stable gene, *TUB*, was used as an internal control. Therefore, the incorrect use of reference genes can lead to the misinterpretation of data. Moreover, the two most stable gene pairs can serve as internal controls in gene expression studies of different flower samples of *R. molle*.

## Conclusion

This is the first study to conduct a systematic exploration of *R. molle* to validate candidate reference genes for qRT-PCR normalization in various tissues or flower samples of different developmental stages. Eleven housekeeping genes were assessed. *EF1-*α, *RPL3*, *18S*, and *ACT5* were identified as optimum internal control genes in different tissues of *R. molle* and different flower samples. Furthermore, expression profiles of *RmPSY* and *RmPDS* were used to validate the suitability of the reference genes selected in this study. The results show that expression profiles normalized by the most stable reference gene (*EF1-*α, *18S*, *RPL3*, *ACT5*) were similar but became obscured when the least stable reference gene (*TUB*) was used. These results present useful information for reliable qRT-PCR data normalization in *R. molle* gene expression studies.

## Author Contributions

Conceived and designed the experiments: ZX and JS. Performed the experiments: ZX. Analyzed the data: ZX, JS, XS, and XL. Revised the final version of the paper: CL, LH, and SC. Approved the final version of the paper: ZX and JS.

## Conflict of Interest Statement

The authors declare that the research was conducted in the absence of any commercial or financial relationships that could be construed as a potential conflict of interest.

## Footnotes

1 http://bioinfo.ut.ee/primer3-0.4.0/primer3/
